# Bolt-Loosening Monitoring Framework Using an Image-Based Deep Learning and Graphical Model

**DOI:** 10.3390/s20123382

**Published:** 2020-06-15

**Authors:** Hai Chien Pham, Quoc-Bao Ta, Jeong-Tae Kim, Duc-Duy Ho, Xuan-Linh Tran, Thanh-Canh Huynh

**Affiliations:** 1 Applied Computational Civil and Structural Engineering Research Group, Faculty of Civil Engineering, Ton Duc Thang University, Ho Chi Minh City 700000, Vietnam; phamhaichien@tdtu.edu.vn; 2 Ocean Engineering Department, Pukyong National University, Busan 48513, Korea; qb.tabao@gmail.com (Q.-B.T.); idis@pknu.ac.kr (J.-T.K.); 3 Faculty of Civil Engineering, Ho Chi Minh City University of Technology (HCMUT), Ho Chi Minh City 700000, Vietnam; hoducduy@hcmut.edu.vn; 4 Vietnam National University, Ho Chi Minh City 700000, Vietnam; 5 Faculty of Civil Engineering, Duy Tan University, Danang 550000, Vietnam; tranxuanlinh@dtu.edu.vn; 6 Center for Construction, Mechanics and Materials, Institute of Research and Development, Duy Tan University, Danang 550000, Vietnam

**Keywords:** structural health monitoring, damage detection, bolted connection, loosened bolts, bolt loosening, looseness detection, deep learning, R-CNN, image processing, Hough transform

## Abstract

In this study, we investigate a novel idea of using synthetic images of bolts which are generated from a graphical model to train a deep learning model for loosened bolt detection. Firstly, a framework for bolt-loosening detection using image-based deep learning and computer graphics is proposed. Next, the feasibility of the proposed framework is demonstrated through the bolt-loosening monitoring of a lab-scaled bolted joint model. For practicality, the proposed idea is evaluated on the real-scale bolted connections of a historical truss bridge in Danang, Vietnam. The results show that the deep learning model trained by the synthesized images can achieve accurate bolt recognitions and looseness detections. The proposed methodology could help to reduce the time and cost associated with the collection of high-quality training data and further accelerate the applicability of vision-based deep learning models trained on synthetic data in practice.

## 1. Introduction

Bolting is one of the most widely used fastening techniques for linking load-bearing members in civil structures. In construction and operation phases, bolts can be subjected to unexpected mechanical shocks and vibrations which consequently cause bolt looseness, threatening the desired load-carrying capacity of bolted connections [1,2,3]. Therefore, detecting loosened bolts in bolted connections is a well-established topic but challenging for realistic large connections that often contain numerous bolts.

In the past few decades, a large number of innovative structural health monitoring (SHM) and damage detection tools based on the vision-based approach have been developed [4,5,6,7]. The vision-based approach refers to the use of images/videos obtained from digital cameras to record damage-sensitive structural information for integrity characterization and damage identification. As compared with the traditional sensor-based SHM, the vision-based approach offers unique advantages. While the sensor-based SHM often requires expensive data acquisition systems [8,9,10,11,12,13], the vision-based approach requires only simple and low-cost setup and operation. Besides this, vision sensors are non-contact, while traditional sensors must be contacted to a target structure, causing degradations in the sensor’s quality under environmental effects [14,15]. Furthermore, the structural information obtained from a vision sensor is not affected by environmental variation, while the signals obtained from a contacted sensor can be significantly affected by environmental changes, which is the main cause of false detections [16,17,18,19]. Lastly, the vision-based approach is suitable for monitoring a large-size structure because an imagery frame can provide the structural responses of certain points concurrently, whereas the traditional sensor-based approach often requires a vast quantity of sensors and measurement channels to cover a large structure.

Besides those advantages, there still exist limitations in the vision-based approach [20]. For instance, the movement of a vision device, varying lighting conditions, fog, and ground vibration are the main causes of the reduction in the quality of captured images. The image resolution and camera costs are also main issues when measuring the structural responses of a large structure at high accuracy. Recent research has focused on developing more advanced and low-cost vision-based techniques for on-site applications with complicated conditions. For examples, Kromanis and Forbes [21] introduced a low-cost robotic camera system which allows collecting high pixel density for the accurate measurement of structural responses; other researchers have developed smartphone-based monitoring systems to measure structural deformation with an inexpensive cost [22].

Recently, several research groups have put their efforts into developing vision-based approaches for loosened bolt assessment [23,24,25,26,27,28]. A pioneered vision-based approach was developed by Park et al. [23]. In their method, digital images are captured from a target bolted connection and passed through a sequence of image processing steps to estimate the bolt rotations and signals of bolt looseness. Ramana et al. [27] introduced an automated vision-based approach which combines the Viola-Jones algorithm with support vector machines. In the same year, Kong et al. [26] developed an image registration-based bolt-loosening detection of steel joints. The idea is to register and align the images of a steel joint before and after a bolt-loosening event through image registration processes, and loosened bolts are detected if there exist differential features in the registration errors. Although Kong’s method achieved promising results, its major limitation lies in the lighting conditions and camera poses, which need to be similar during an inspection.

The recent success of deep learning in various fields has opened a new paradigm for civil structure monitoring and inspection [7,29]. The robustness and practicality of the traditional vision-based damage identification has been also enhanced by deep learning. Azimi and Pekcan [30] developed a deep learning-based SHM approach using extremely compressed vibration data. The method was experimentally verified on several benchmark structural systems and showed promising prospects for damage identification and localization in realistic large-scale structures. Physics-informed learning, which is an integration of prior knowledge into the deep learning process for augmenting the insufficient training dataset, has raised as a potential solution to enhance the generalizability of deep learning models in a real-world environment [31]. Kim and Cho [32] developed an automated deep learning-based concrete crack detection method which has high adaptability for crack inspection in on-site structures. However, cracks on on-site concrete surfaces are sometimes interfered with by other surrounding pathologies, making the automatic mapping of cracking patterns more challenging. To deal with such on-site situations, Bruno et al. [33] developed a supper cluster-crack method using hyper-spectral image processing that can detect realistic cracking patterns on concrete surfaces with biological stains. The above studies have demonstrated not only the feasibility but also the applicability of vision-based deep learning approaches for on-site structural monitoring.

Researchers have put their efforts into developing vision-based deep learning approaches for loosened bolt detection in bolted joints. Zhao et al. [34] used a trained deep neural network to recognize the rotational features of a single bolt’s surface. Nonetheless, this method has less practicality in monitoring realistic connections, which often contain hundreds of bolts. Wang et al. [35] designed another deep learning-based method for bolt looseness detection. In their method, the number “5” is marked on one bolt of the flange connection for bolt detection through deep learning; then, image processing techniques are used to detect the bolt rotation. However, the marking process of the number “5” on a bolt will cause inconveniences when applying for in situ inaccessible connections. More recently, Huynh et al. [36] developed a vision-based bolt-loosening detection method using a region-based convolutional neural network (R-CNN) and image processing. The proposed idea is to use a trained R-CNN model to recognize bolts in the connection and image processing to detect loosened bolts.

However, the perception capacity of a vision-based deep learning model is dependent on the number of available datasets. A trained model can perform unsuccessfully if the extracted features from the input data are significantly different from the training data. The limited number of labelled datasets will cause difficulties to generalize a trained deep learning model across a wide variety of structures and environmental conditions [7]. Each civil structure is unique, therefore making automated deep learning-based damage detection more challenging. To advance the capability and robustness of the deep learning models for automated monitoring and inspection, the issue of the limited quantity of training datasets must be overcome. Recent advances in computer graphics allow researchers to build realistic virtual models which can be used to generate training graphic datasets with desired conditions. Deep learning models trained on the graphic datasets have recently shown a promising performance with realistic datasets [37,38]. The use of virtual graphic models can provide many advantages, including (1) fully-automated labelling the objects at both the image and pixel levels [39]; (2) providing a high-quality testbed for testing vision-based algorithms with repeated conditions; (3) easily simulating different environmental conditions (e.g., lighting, corrosion), and studying algorithms with different posing parameters [7].

In this study, we investigate a novel idea to use synthetic datasets to train a vision-based deep learning model for loosened bolt detection. The study aims at reducing the time and cost associated with the collection of high-quality datasets and further accelerate the applicability of vision-based deep learning models trained on synthetic data in practice. At first, a bolt-loosening detection framework using image-based deep learning and a graphical model is proposed. Next, the feasibility of the proposed methodology is demonstrated via the bolt-loosening monitoring of a lab-scaled joint model. For practicality, the proposed idea is consequently evaluated on the real-scale bolted connections of a historical bridge in Danang, Vietnam.

## 2. Bolt-Loosening Detection Framework

### 2.1. Overview of the Framework

**Figure 1:**
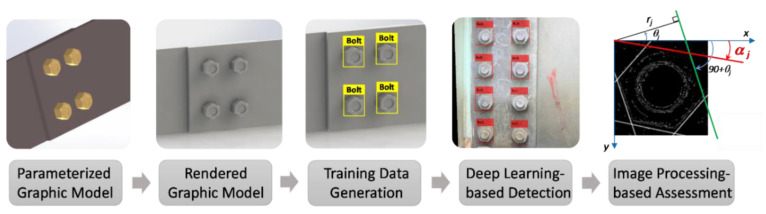
Framework for bolt-loosening monitoring through image-based deep learning and a graphical model.

A bolt-loosening detection framework using image-based deep learning and a graphical model is proposed in Figure 1. The methodology is to use the graphical model to train a deep learning model for bolt detection and image processing to estimate the rotation of bolts for bolt-loosening detection. Overall, the proposed framework has five steps: (1) create a parameterized graphic model of a representative connection using a 3D simulation tool; (2) render the parameterized graphic model by applying realistic material properties; (3) generate graphic images from the rendered graphic model and create a training graphical databank; (4) train a deep learning model for bolt detection; and, finally, (5) estimate the rotational angles of detected bolts using image processing techniques.

### 2.2. Image-Based Deep Learning Method for Bolt-Loosening Detection

**Figure 2:**
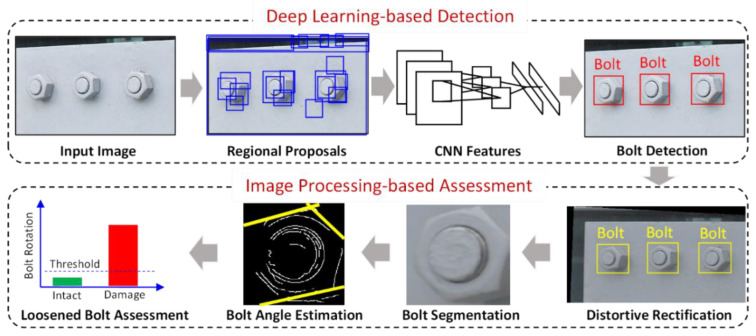
The image-based deep learning model for bolt-loosening detection.

In this study, an image-based deep learning method for loosened bolt detection developed by Huynh et al. [36] was selected for the proposed framework. The method was particularly designed for large-scale bolted connections in civil infrastructure which often consist of numerous bolts. As depicted in Figure 2, the method has two phases: deep learning-based bolt detection (Phase I) and image processing-based bolt-loosening assessment (Phase II), which are described in detail in Section 2.2.1 and Section 2.2.2, respectively.

#### 2.2.1. Phase I: Deep Learning-Based Bolt Detection

**Figure 3:**
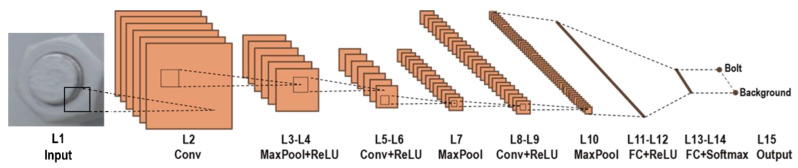
The overall structure of the bolt classifier consisting of 15 convolutional neural network (CNN) layers [36].

The deep learning-based bolt detection method is based on the R-CNN model [36]. The idea of the R-CNN model is based on the extraction of object proposals from the input image using a selective search method and the classification of the extracted proposals using a CNN model. Phase I has four major steps: (a) a digital image of a bolted joint is fed into the R-CNN model; (b) from the input image, object proposals are identified using the selective search method [40]; (c) the identified object proposals are re-sized and passed through the CNN model to calculate the feature vectors; and (d) each identified object proposal is classified into bolt or background (not bolt) by using the computed CNN features. The CNN model is configured with 15 layers, as sketched in Figure 3. Briefly, the model has an Input layer, three Conv (convolutional) layers, three MaxPool (max pooling) layers, four ReLU (rectified linear unit) layers, two FC (fully connected) layers, a Softmax layer, and an Output layer. The details of the CNN layers are presented in [36].

#### 2.2.2. Phase II: Image Processing-Based Bolt-Loosening Assessment

After the deep learning-based bolt detection, Phase II is performed to detect the loosened bolts in the bolted connection. Phase II is performed in four main steps: (a) the perspective distortion of the input image is corrected by the homography; (b) the detected bolts are cropped into sub-images; (c) the bolt angles of the cropped images are estimated by the Hough transform; and, finally, (d) loosened bolts are identified by computing the rotations of the detected bolts.

Homography-Based Perspective Rectification

The perspective rectification algorithm is based on the homography, which is a projective transformation [25,41]. With a known homography matrix H, a point p*_j_* = (*u_j_*, *v_j_*, 1) in an image plane can be correspondingly transformed to a point q*_j_* = (*x_j_*, *y_j_*, 1) in another plane, as expressed in Equation (1).

(1) $$p_{j}={\mathrm{Hq}}_{j}\;\mathrm{where}\;H=\left[\begin{array}{ccc} h_{11} & h_{12} & h_{13}\\ h_{21} & h_{22} & h_{23}\\ h_{31} & h_{32} & 1 \end{array}\right]$$

The matrix H has eight independent components, which can be determined using four points in the reference image and those in the distorted image [36]. By inversion of the matrix H, the distorted connection image is converted to a new image (i.e., a rectified image).

Hough Transform for Bolt Angle Estimation

The Hough transform (HT)-based bolt angle estimation algorithm has four steps [23]: (a) the edges of a bolt are detected by the Canny method [42]; (b) the detected edges are transformed into the Hough space; (c) from a store accumulator of the detected edges, the two realistic edges are selected; and (d) the formulas of the selected edges are extracted for bolt angle estimation.

The main idea is to map single points from the image space (*x, y*) to the Hough space, where a line is represented by two parameters *θ* ∈ [−π, π] and *r* ≥ 0, as follows:

(2) r=xcosθ+ysinθ

where *θ* and *r* are the angle of the line and the distance from the line to the origin, respectively. A point in the image space shall be converted to a curve in the Hough space using Equation (2). The converted curves may intercross at some points in the Hough space, and the points where the curves mostly intercross potentially corresponds to the realistic edges of the bolt.

**Figure 4:**
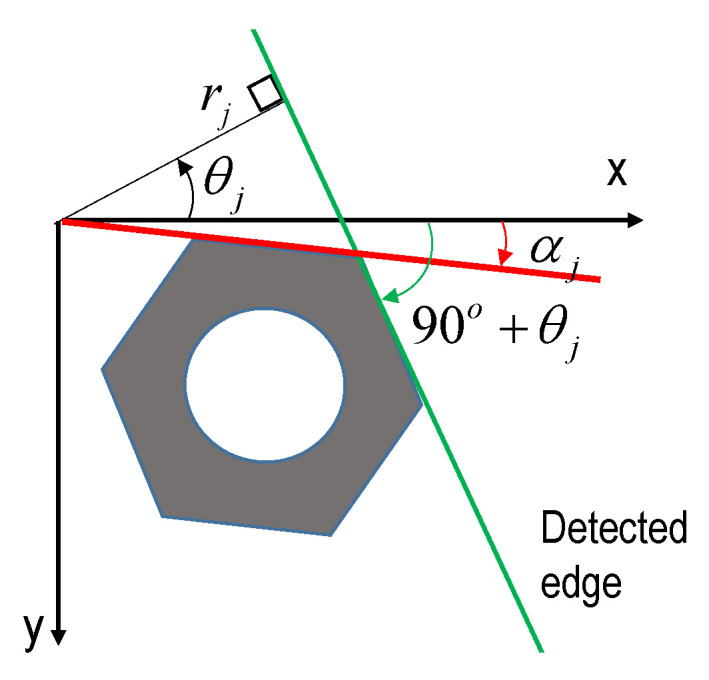
Estimation method for the bolt angle.

The angle of the bolt’s edges detected by the HT is defined for a hexagon bolt in Figure 4. The *j*th realistic bolt edge, represented by (*θ_j_*, *r_j_*), has an angle with the horizontal direction of *θ_j_* + 90^o^. This bolt angle can be represented in 0°–60° as:

(3) $$\alpha_{j}=\mathrm{mod}\left[(\theta_{j}+90^{o})/60^{o}\right]$$

where *α_j_* is considered as the *j*th bolt angle (see Figure 4); mod [.] is an operator to compute the rest of dividing. With k realistic edges, the bolt angle is finally computed as:

(4) $$\alpha =\frac{1}{k}\sum_{j=1}^{k}\alpha_{j}$$

where *α* is the averaged angle.

Loosened Bolt Detection Using Upper Control Limit

For detecting a loosened bolt, the bolt rotation Δα is calculated as follows:

(5) $$\Delta \alpha =\alpha^{*}-\alpha$$

where the terms α^*^ and *α* are the present and the reference bolt angles, respectively. The absolute value of the bolt rotation |Δα| is then compared with the upper control limit (UCL) threshold as:

(6) |Δα|>UCL

The threshold *UCL* is estimated by three standard deviations of the mean (a confidence level of 99.7%) [23,36]. If the condition in Equation (6) is fulfilled, the monitored bolt is classified as a loosened bolt, or else the bolt is not loosened.

### 2.3. Training Deep Learning for Bolt Detection by Graphical Model

#### 2.3.1. Constructing Graphical Databank for Bolt Detection

**Figure 5:**
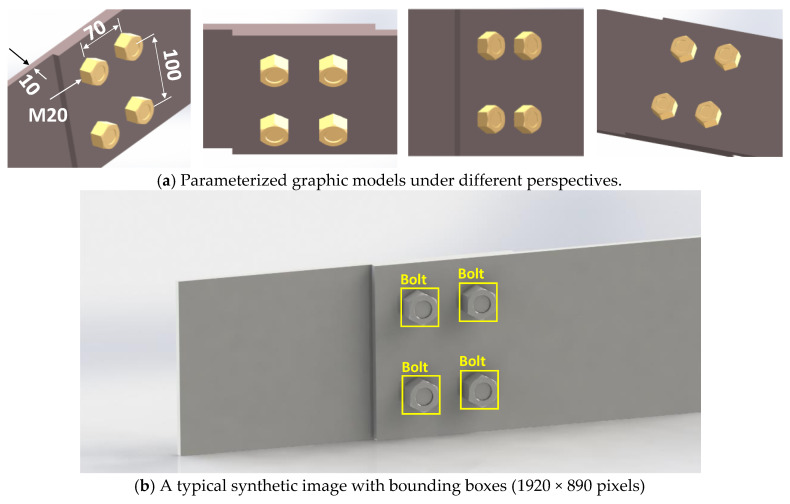
Synthetic data for training the deep learning-based bolt detection model.

As described in Section 2.1, the functionality of the graphical model is to synthesize the training data for bolt detection through deep learning. A 3D modelling tool, Solidworks, was used to create the graphical model of bolts. A single lap joint was selected as a representative bolted connection model, as shown in Figure 5a. Two steel plates are joined by four standard hexagon bolts (M20 mm) and nuts, which are positioned in a 2 × 2 array. The horizontal distance between the bolts’ center is 70 mm, and the vertical distance between the bolts’ center is 100 mm. The steel plates have a thickness of 10 mm, a height of 140 mm, and a length of 400 mm. It is noted that the bolt spacing is not an issue, but the bolt types. The deep learning model should be retrained if the target structure has different bolt types. However, the generation of the data of new bolt types for a new training task can be achieved easily by reconfiguring the bolt types in the graphical model through Solidworks. In this work, the hexagon bolt type was selected to evaluate the proposed framework.

The parameterized graphic model was then rendered by applying lighting conditions and material properties, as shown in Figure 5b. It is supposed that the bolted connection is painted by a grey colour. After rendering, it is shown that the rendered model looks realistic, with lighting effects and shadows on it. The synthetic images were captured. To construct a training databank for deep learning, 99 images were captured under various scales and perspectives and then manually labelled with ground-truth bounding boxes (indicating bolts). Figure 5b shows a typical training image with a resolution of 1920 × 890 pixels.

#### 2.3.2. Training Deep Learning Model for Bolt Detection

The RCNN-based bolt detection model was trained using transfer learning, which is based on a pre-training process to reduce the training time and convergence for a new detection task [43]. At first, the R-CNN model was pre-trained using 50,000 images of the CIFAR-10 dataset, which has ten classes: airplane, automobile, bird, cat, deer, dog, frog, horse, ship, and truck [44]. Herein, the algorithm for pre-training was the stochastic gradient descent with momentum (SGDM). The parameters of the SGDM were set as the same as those in the previous work [36]. The R-CNN model achieved an accuracy of 74% after training epochs (a learning rate of 0.001, a learning rate drop of 8 epochs, and a mini-batch size of 128).

**Figure 6:**
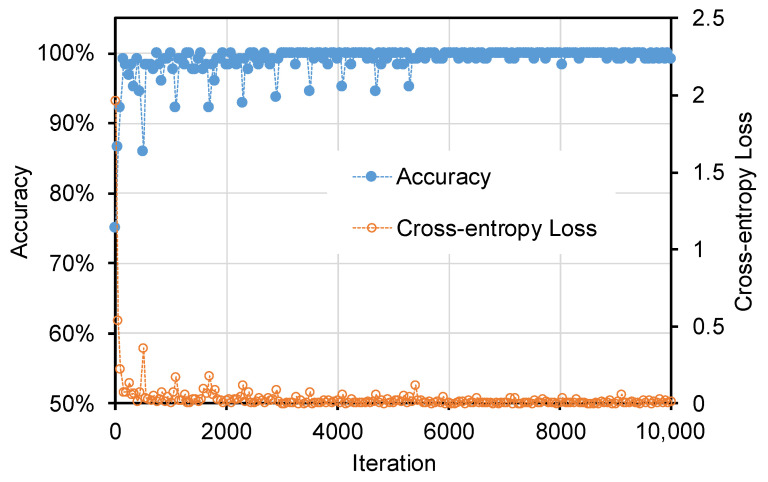
Accuracy and cross-entropy loss of the bolt detector during the training process.

Next, the bolt detector was trained using the training synthetic databank generated in Section 2.3.1. The SGDM training algorithm was also used. Regional proposals having the intersection over union equal or higher than 0.8 were defined as positive training samples, while negative ones were extraneous. The training with the synthetic databank was performed in 668 s with 10,000 iterations. The parameters of the training algorithm were set as similar to those presented in the previous work [36]. Figure 6 shows the training progress of the R-CNN model. The model achieved an accuracy of 99 % at the 10,000th iteration. The corresponding cross-entropy loss was dropped to 0.0058 at the final iteration.

It is worth noting that the graphic model can be easily rendered with different material properties and environmental conditions and also reconfigured to generate additional graphic images for a new training task. Therefore, the use of the graphical model can help to overcome difficulties in the generalization of a deep learning model over different target structures and environmental conditions and further reduce the time and cost associated with the creation of the vast quantities of high-quality labelled data.

#### 2.3.3. Testing Deep Learning Model for Bolt Detection

**Figure 7:**
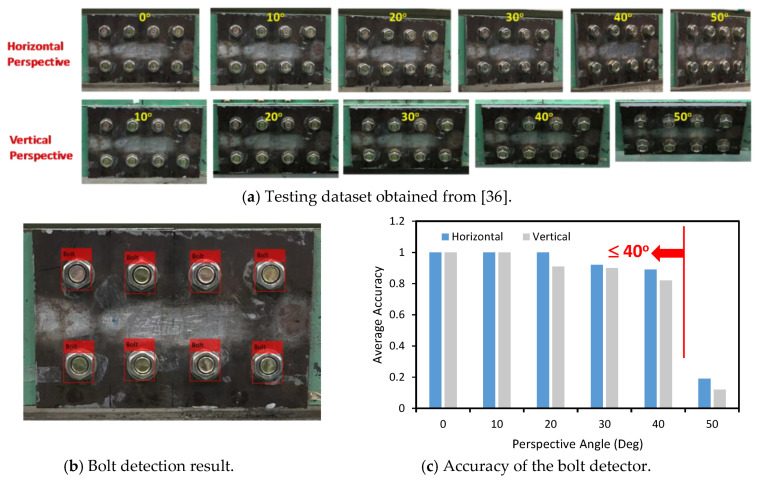
Testing the bolt detector on an existing realistic dataset.

The performance of the trained deep learning model was tested on a realistic dataset obtained from an existing publication [36]. The testing dataset is composed of 88 labelled images of a lab-scaled girder connection of eight bolts (M20, Korean standard), as shown in Figure 7a. A detailed description of the testing dataset can be found in [36].

Figure 7b shows that all the bolts in a connection image are well detected. As can be seen in Figure 7c, the precision of the bolt detector was computed for different horizontal and vertical perspective angles. The bolt detection has an average accuracy of between 90% and 100% for the perspective angle of 10°–30°. This accuracy decreased to 89% and 82% as the horizontal/vertical perspectives reached 40°. When the horizontal distortion increased to 50°, the accuracy dropped to 19%. For the vertical perspective, the precision was only 12% at the distortive angle of 50°. It is shown that the perspective angle of a connection image should be equal to or less than 40° to ensure the reliability of the bolt detector. As compared with the bolt detector trained by the realistic data in [36], the bolt detector trained by the synthetic data can achieve comparably good detection results.

## 3. Loosened Bolt Identification of Lab-Scaled Bolted Connection

### 3.1. Experimental Setup of Bolted Connection in Laboratory

**Figure 8:**
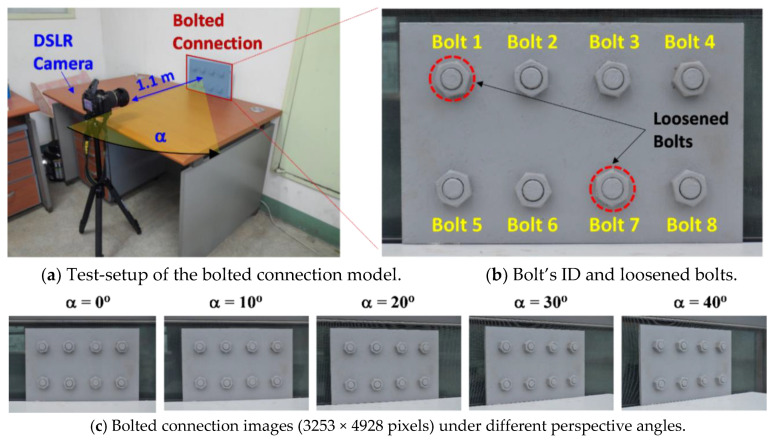
Experimental setup of lab-scaled bolted connection model.

The proposed framework was evaluated on a lab-scaled bolted connection model. Figure 8a shows the experimental setup of the laboratory test. The bolted joint model has a steel splice plate and eight pairs of bolts and nuts (M20, Korean standard); see Figure 8b. The splice plate has a length of 310 mm, a height of 200 mm, and a thickness of 10 mm. The bolted connection model was coated by grey anticorrosive paint. A digital camera, a Nikon D7000 model with a Tamron 17–50 mm/f2.8 lens and a 3253 × 4928 pixel resolution, was used to capture the connection images. The camera was fixed with a tripod with a distance of 1.1 m in front of the test connection, as shown in Figure 8a.

To evaluate the accuracy of the proposed framework, the bolt-loosening test was performed. Figure 8b shows an image of the bolted connection for the intact case (before bolt-loosening). Among eight bolts, Bolt 1 and Bolt 7 were selected to be loosened, as indicated in Figure 8b. Six bolt-loosening cases (i.e., Damages 1–6) were simulated for the test connection, as described in Table 1. Firstly, Bolt 1 was respectively loosened by 5°, 15°, and 45° to simulate Damages 1–3. Next, Bolt 7 was loosened by 12°, 23°, and 57° to simulate Damages 4–6, respectively. For the intact case and each of the damaged cases, ten connection images were captured. In total, 70 images of the connection were captured. For comparison, the bolt angles before and after a loosening event were accurately measured by a digital goniometer.

To evaluate the effect of perspective distortion on the performance of the proposed framework, a varying perspective test was conducted. Accordingly, the perspective angle α (see Figure 8a) was changed from 0° to 10°, 20°, 30°, and 40°. Ten images of the connection were captured for each of the perspective angles, as shown in Figure 8c. In total, 50 connection images were captured.

### 3.2. Accuracy of the Proposed Framework

**Figure 9:**
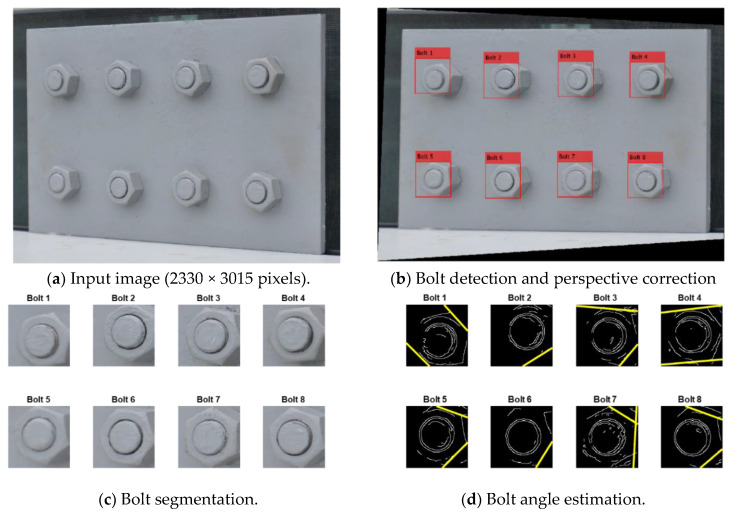
The representative results of the lab-scaled connection image with a 30° horizontal perspective.

**Figure 10:**
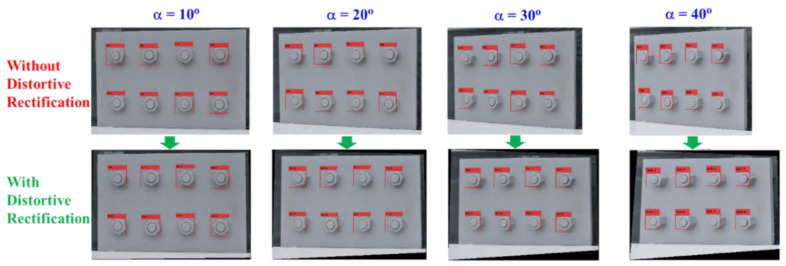
Detection of bolts in the distorted and undistorted images of the lab-scaled connection model.

The varying perspective test was used to evaluate the performance of the proposed framework under various perspective angles. Figure 9 shows the representative results of bolt identification and bolt angle calculation for a connection image captured under a 30° horizontal perspective distortion. It is shown from Figure 9a that all the bolts in the image were successfully detected by the deep learning-based bolt detector. Figure 9b shows that the perspective angle of the distorted image was well rectified. After bolt detection, the identified bolts were sorted and named as Bolt 1–Bolt 8. As noted from Figure 7, for a perspective angle of 30°, the accuracy of the bolt detector was about 90%. Thus, some small regions of nuts can be missed in some detected bolts, as extracted in Figure 9c. Afterwards, the angles of all the bolts were estimated by the HT algorithm. As shown in Figure 9d, the two strongest edges were well identified for all the bolts, except Bolt 2 and Bolt 6, for which single edges were detected. The bolt detection results for the images with and without perspective correction are shown in Figure 10. The bolts were detected with good accuracy in both images, even when the perspective angle reached 40°. This result validated the deep learning model trained on the synthetic data for bolt recognitions.

**Figure 11:**
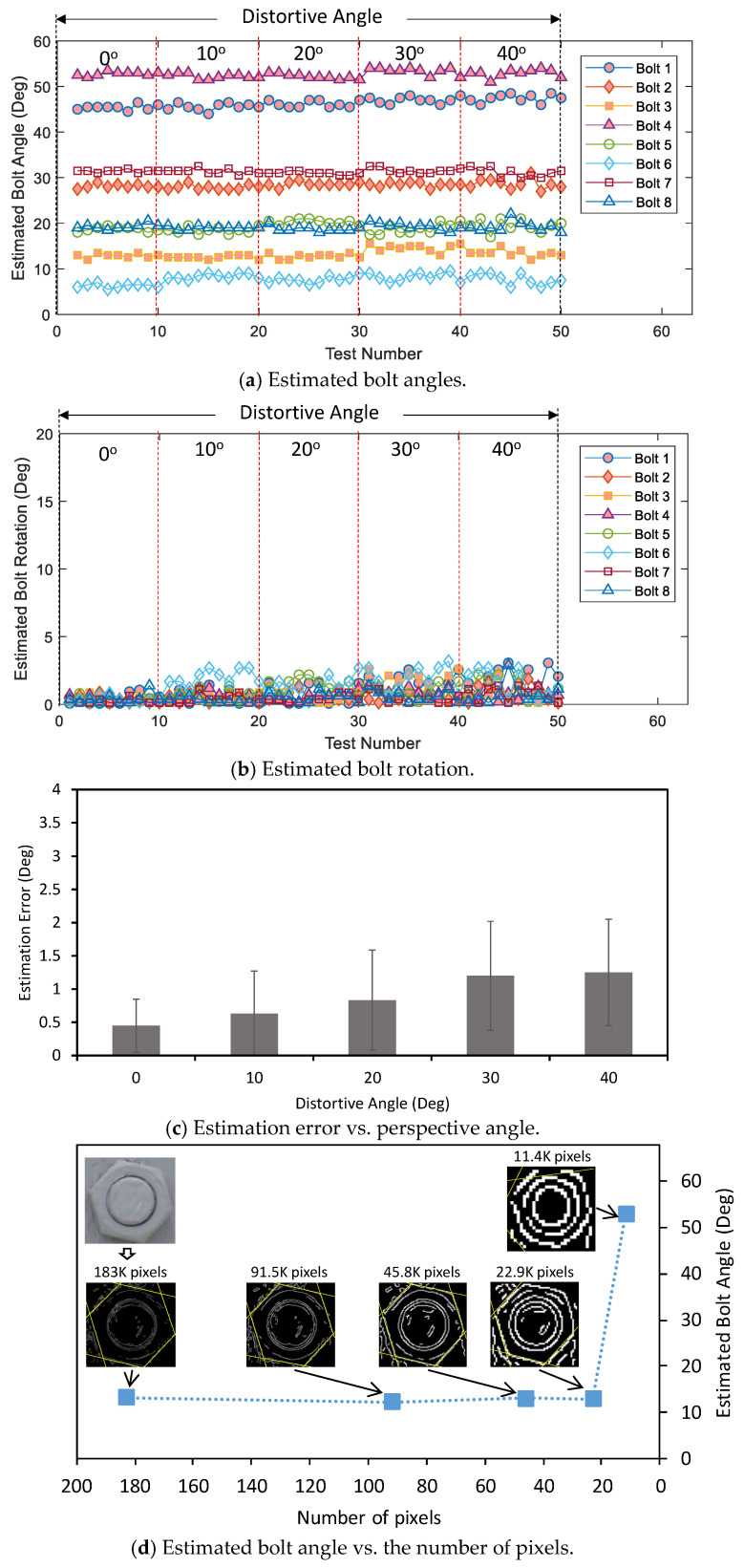
Accuracy of the proposed framework under different perspectives.

Figure 11a shows the estimated bolt angle along with the test number under the intact state. There exists strong consistency in the estimation of bolt angles. The bolt rotation was computed for different perspective angles under the intact state, as shown in Figure 11b. The estimated bolt rotation was ignorable when there was no effect of perspective distortion (α = 0°); however, the estimation exhibited a higher deviation when the distortive angle of the image raised. The estimation error of bolt rotation was analyzed concerning the perspective angle in Figure 11c. The estimation error became more significant as the capturing angle increased; particularly, the error was only 0.45° ± 0.4° at the perspective angle of 0° but 1.25° ± 0.8° at the perspective angle of 40°.

We have also analyzed the effect of the number of pixels on the angle calculation of a single bolt, as shown in Figure 11d. It is noted that a single bolt (in a front view) consists of about 183 K pixels. It is shown the estimated bolt angle negligibly varied when the number of pixels decreased from 183 to 22.9 K, but rapidly varied as the number of pixels reduced from 22.9 to 11.4 K. To ensure the applicability of the proposed method, the number of pixels of a single bolt should not be less than 22.9 K.

### 3.3. Detection of Loosened Bolts

The bolt-loosening test was used to analyze the accuracy of the proposed framework for the detection of loosened bolts. Figure 12a shows the estimated rotational angles of Bolt 1–Bolt 8 for the undamaged case (i.e., intact case) and Damages 1–6. It is shown that for the images 1–10 (i.e., the undamaged case), the rotational angles of the bolts were below the UCL of 1.54°, indicating that all the bolts were not loosened. Nonetheless, for the images 11–20 (i.e., Damage 1), the rotational angle of Bolt 1 was above the threshold, suggesting that Bolt 1 was loosened. The images 21–40 (i.e., Damages 2–3) showed the increases in the bolt-loosening severity of Bolt 1. For the images 41–50 (Damage 4), the rotational angle of Bolt 7 was above the UCL level, revealing that Bolt 7 was loosened. The images 51–50 (Damages 5–6) showed the changes in the rotational severity of Bolt 7. Conclusively, all damage cases in the bolted joint were well alarmed and the loosened bolts were well detected, even with only 5° bolt rotation (Damage 1).

**Figure 12:**
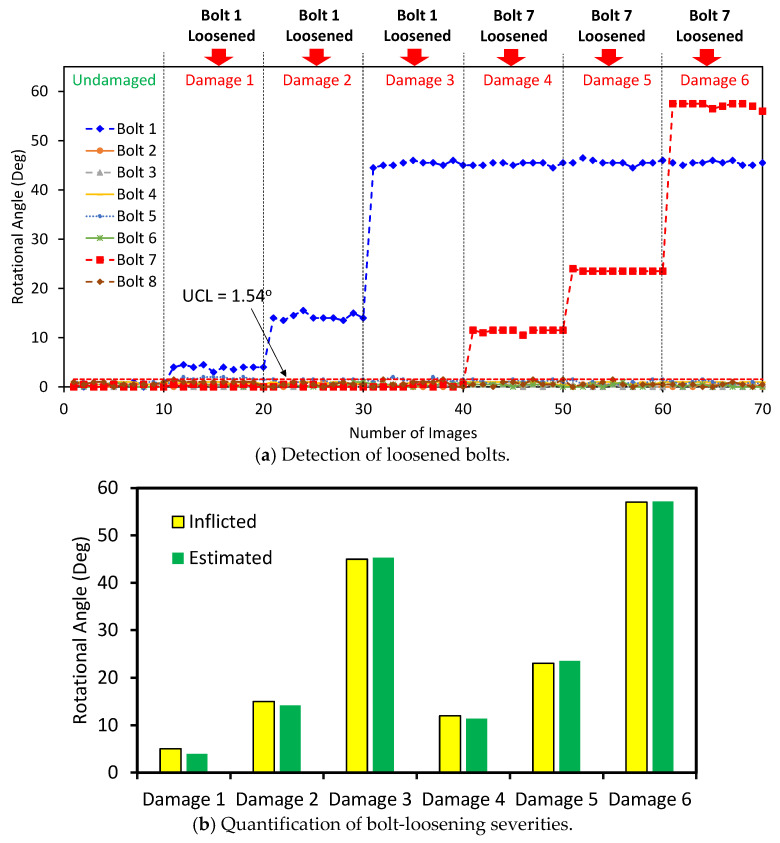
Bolt-loosening detection in the lab-scaled bolted connection model.

Figure 12b compares the bolt-loosening severities estimated by the proposed framework with the inflicted ones. It is observed that the estimated bolt rotations well agreed with the inflicted ones for the six damage cases, suggesting the reliability of the proposed framework for bolt-loosening detection.

## 4. Monitoring of Bolted Connections of a Historic Truss Bridge

### 4.1. Field Tests on the Historic Truss Bridge

**Figure 13:**
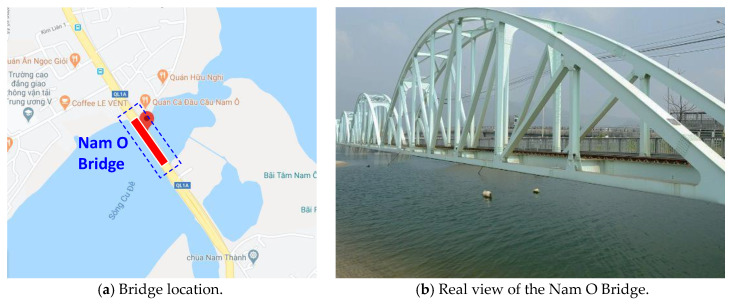
The Nam O Bridge in Da Nang (Vietnam) as a test structure.

For the practicality, the proposed framework was evaluated on the Nam O Bridge in Da Nang City, Vietnam, as shown in Figure 13a. The Nam O Bridge is a historical truss bridge consisting of many large bolted joints, as shown in Figure 13b. The bridge was constructed before 1975 (the year of the reunification of Vietnam) and maintained several times. The monitoring of the structural condition of the bridge connections is essential to ensure their safety and serviceability. Herein, we used a digital camera to capture the images of several bolted connections (a resolution of 4032 × 3024 pixels; AF f/2.8; a focal length of 7 mm). The camera distance was within 1.0 m–1.5 m. The connection images were input into the proposed framework for bolt-loosening monitoring. Two representatives—an inclined joint and a repaired joint—were selected to evaluate the in situ performance of the proposed framework. The bolt-loosening monitoring results of the representative joints are presented in Section 4.2 and Section 4.3.

### 4.2. Bolt-Angle Monitoring of Inclined Bolted Connection

**Figure 14:**
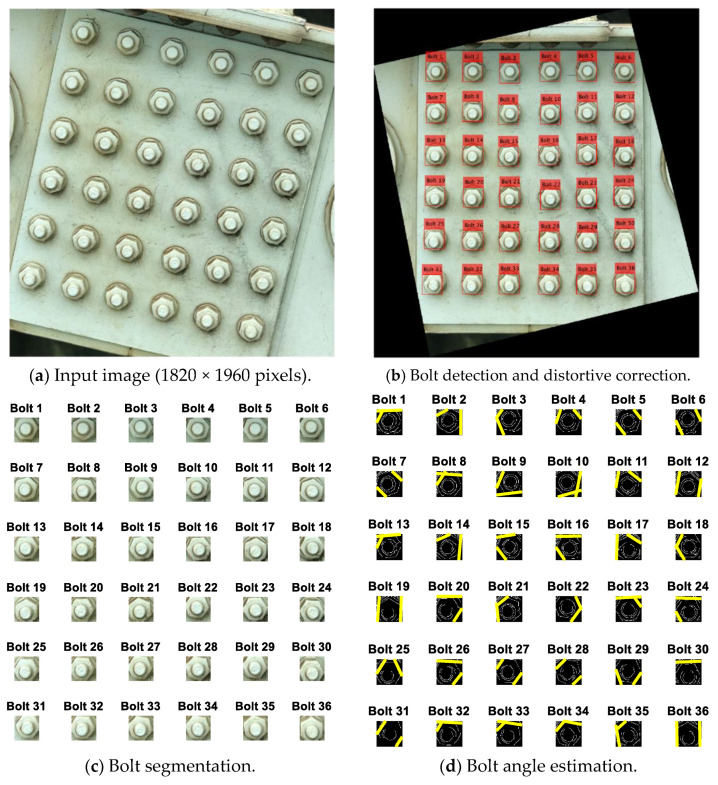
Bolt angle estimation of the included bolted connection.

Figure 14 shows the estimation of the as-of-now bolt angles of the inclined bolted connection. The test connection is composed of 36 bolts, as shown in Figure 14a. The perspective of the captured image was corrected by the homography-based distortion rectification algorithm, as shown in Figure 14b. It is shown that the 36 bolts were well identified in the image of the inclined bolted connection. Figure 14c shows the 36 cropped images of the identified bolts (i.e., Bolt 1–Bolt 36). The bolt angles were estimated by the HT algorithm. It is shown in Figure 14d that the two realistic edges were well-identified for all the bolts except Bolt 30, for which a single edge was detected.

**Figure 15:**
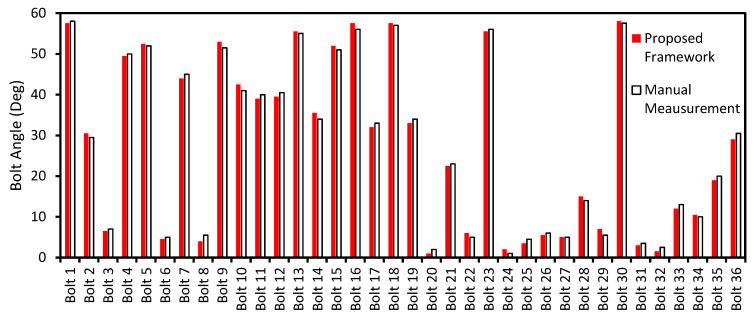
The accuracy of the bolt angle estimation of the inclined bolted connection.

Figure 15 confirms the performance of the proposed framework for the estimation of the as-of-now bolt angle. It is shown that the estimated angles of the 36 bolts by the proposed method well agreed with the manual measurement of a digital goniometer. The root-mean-square-error of the estimation was only 0.97°. The result confirms the accuracy of the proposed framework for bolt-rotation monitoring.

### 4.3. Bolt-Angle Monitoring of Repaired Bolted Connection

**Figure 16:**
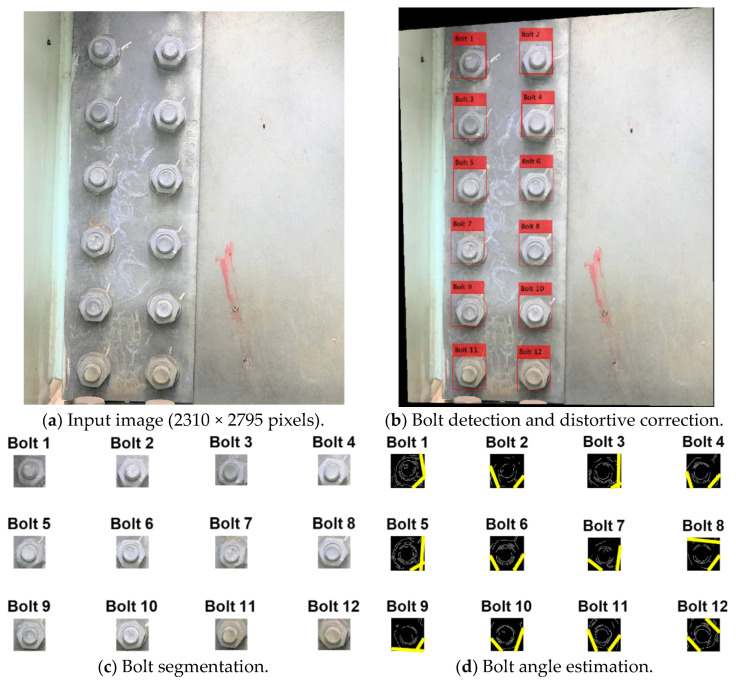
Results of the vision-based bolt-loosening monitoring method.

**Figure 17:**
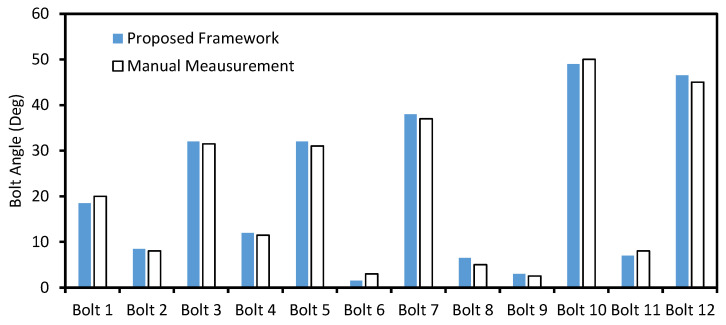
The accuracy of the bolt angle estimation of the repaired bolted connection.

Next, the bolt angles of a repaired bolted connection were estimated. As shown in Figure 16a, the connection is composed of 12 bolts. The perspective angle of the captured image was corrected, as depicted in Figure 16b. It is shown that the 12 bolts were well identified in the image of the test bolted joint. Figure 16c shows the 12 cropped images of the identified bolts (i.e., Bolt 1–Bolt 12). Then, the bolts’ edges were identified, as shown in Figure 16d. The accuracy of the proposed framework was compared with the manual measurement in Figure 17. It is observed that the two methods exhibited similar estimation results, for which the root-mean-square-error was only 1.08°. This result evidences the accuracy of the proposed framework for the bolt-loosening monitoring of realistic bolted joints.

## 5. Conclusions

In this study, we investigated a novel idea using synthetic data to train a deep learning model for bolt-loosening detection. Firstly, a bolt-loosening monitoring framework using an image-based deep learning model trained by computer graphics was presented. Secondly, the feasibility of the proposed idea was evaluated via the bolt-loosening monitoring of a lab-scaled bolted connection. Thirdly, for the in situ applicability, the proposed idea was evaluated on a historical truss bridge in Danang, Vietnam.

At least three concluding remarks can be drawn as follows: (1) Both the laboratory and field tests showed that the deep learning model trained by the synthesized images can provide good bolt recognition and bolt angle estimation. (2) The laboratory test demonstrated the feasibility of the proposed idea of training a deep learning model on graphical data for loosened bolt detection. The estimation error of bolt loosening increased along with the perspective angle; the error was ignorable for small perspective distortions and 1.25° ± 0.8° for the perspective angle of 40°. The number of pixels of the image of a single bolt should not be less than 22.9 K to ensure the accuracy of the bolt angle calculation. (3) The field test results evidenced the practicality of the proposed framework using deep learning and a graphical model for large joint monitoring. The as-of-now bolt angles of the representative bolted joints were estimated with high accuracy.

The present study opened an alternative strategy to synthesize training databanks with saved times and costs. The graphic model can be easily reconfigured to generate additional high-quality images for a new training task. Besides, the results of this study further demonstrate the use of deep learning models trained on the graphical dataset to work with a real dataset. The presented methodology is promising to be integrated with the devices carrying digital cameras (e.g., drones, robotic cameras, and smartphone cameras) to carry out a vision-based bolt-loosening assessment on real-world structures.

As the future works, an adaptive Canny edge detector shall be implemented to the proposed framework to improve the detectability of the bolt edges. Besides this, some bolt rotations shall be artificially simulated in the images of the full-scale structure for a better demonstration of the practicality of the proposed methodology. Lastly, a detailed valuation of the time and cost related to the collection of high-quality training data by the graphical model shall be investigated.

## Figures and Tables

**Table 1:** Bolt-loosening test of lab-scaled bolted connection model.

Case	Damage 1	Damage 2	Damage 3	Damage 4	Damage 5	Damage 6
Loosened Bolt	Bolt 1	Bolt 1	Bolt 1	Bolt 7	Bolt 7	Bolt 7
Loosening Angle	5°	15°	45°	12°	23°	57°
