# Correction: Pham et al. Bolt-Loosening Monitoring Framework Using an Image-Based Deep Learning and Graphical Model. *Sensors* 2020, *20*, 3382

**DOI:** 10.3390/s21165280

**Published:** 2021-08-05

**Authors:** Hai Chien Pham, Quoc-Bao Ta, Jeong-Tae Kim, Duc-Duy Ho, Xuan-Linh Tran, Thanh-Canh Huynh

**Affiliations:** 1 Applied Computational Civil and Structural Engineering Research Group, Faculty of Civil Engineering, Ton Duc Thang University, Ho Chi Minh City 700000, Vietnam; phamhaichien@tdtu.edu.vn; 2 Ocean Engineering Department, Pukyong National University, Busan 48513, Korea; qb.tabao@gmail.com (Q.-B.T.); idis@pknu.ac.kr (J.-T.K.); 3 Faculty of Civil Engineering, Ho Chi Minh City University of Technology (HCMUT), Ho Chi Minh City 700000, Vietnam; hoducduy@hcmut.edu.vn; 4 Vietnam National University, Ho Chi Minh City 700000, Vietnam; 5 Faculty of Civil Engineering, Duy Tan University, Danang 550000, Vietnam; tranxuanlinh@dtu.edu.vn; 6 Center for Construction, Mechanics and Materials, Institute of Research and Development, Duy Tan University, Danang 550000, Vietnam

The authors wish to make the following correction to this paper [1].

Figure 13b should be replaced by the following picture:


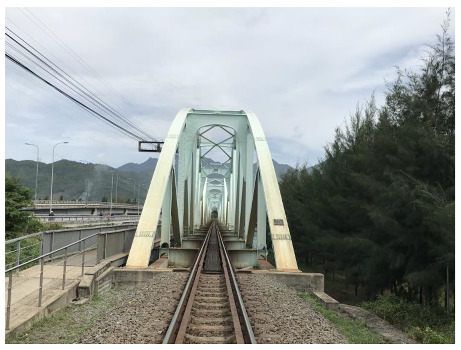


The authors apologize for any inconvenience caused and state that the scientific conclusions are unaffected. The original article has been updated.
